# Twelve years of GWAS discoveries for osteoporosis and related traits: advances, challenges and applications

**DOI:** 10.1038/s41413-021-00143-3

**Published:** 2021-04-29

**Authors:** Xiaowei Zhu, Weiyang Bai, Houfeng Zheng

**Affiliations:** 1 Diseases & Population (DaP) Geninfo Lab, School of Life Sciences, Westlake University, 18 Shilongshan Road, Hangzhou, China; 2 Institute of Basic Medical Sciences, Westlake Institute for Advanced Study, 18 Shilongshan Road, Hangzhou, China; 3 School of Life Sciences, Fudan University, Shanghai, China; 4 Westlake Laboratory of Life Sciences and Biomedicine, Hangzhou, Zhejiang China

**Keywords:** Calcium and phosphate metabolic disorders, Osteopetrosis

## Abstract

Osteoporosis is a common skeletal disease, affecting ~200 million people around the world. As a complex disease, osteoporosis is influenced by many factors, including diet (e.g. calcium and protein intake), physical activity, endocrine status, coexisting diseases and genetic factors. In this review, we first summarize the discovery from genome-wide association studies (GWASs) in the bone field in the last 12 years. To date, GWASs and meta-analyses have discovered hundreds of loci that are associated with bone mineral density (BMD), osteoporosis, and osteoporotic fractures. However, the GWAS approach has sometimes been criticized because of the small effect size of the discovered variants and the mystery of missing heritability, these two questions could be partially explained by the newly raised conceptual models, such as omnigenic model and natural selection. Finally, we introduce the clinical use of GWAS findings in the bone field, such as the identification of causal clinical risk factors, the development of drug targets and disease prediction. Despite the fruitful GWAS discoveries in the bone field, most of these GWAS participants were of European descent, and more genetic studies should be carried out in other ethnic populations to benefit disease prediction in the corresponding population.

## Introduction

Osteoporosis is a common skeletal disease affecting ~200 million people around the world; it is characterized by decreased bone density, bone microstructural damage and a consequent increase in bone fragility.^1,2^ Nearly 22 million women and 5.5 million men were estimated to have osteoporosis in Europe^3^ and 10 million in the United States, and this number continues to rise.^4^ In China, ~83.9 million people are estimated to suffer from osteoporosis, and this number, including osteopenia, should increase to ~212 million people by 2050.^5^ Bone fragility is a poor outcome of osteoporosis, where long-term therapy and medical management are needed, especially in elderly individuals.^1^ By 2050, it is estimated that ~51.1% of worldwide hip fracture cases will be from Asia.^6^ Accordingly, the burden of treatment for osteoporosis and osteoporotic fractures has been rising very rapidly, with an annual cost of $17 billion to treat fractures in the United States.^4,7^ In China, ~2.33 million osteoporotic fractures occurred in 2010, costing $9.45 billion, and the annual costs are estimated to double by 2035.^8^ Therefore, as aging-related diseases, osteoporosis and osteoporotic fracture inflict a substantial economic, social, and clinical burden.

Osteoporosis, as a complex disease, is influenced by many factors, including diet (calcium and protein intake), physical activity, endocrine status, coexisting diseases, and genetic factors.^1^ Osteoporosis is mainly characterized by low bone mineral density (BMD), which is highly heritable, with heritability ranging from 50% to 80%.^2,9^ To date, genome-wide association studies (GWASs) (Supplemental Note Box 1) and meta-analyses have discovered many loci that are associated with BMD, osteoporosis, and osteoporotic fractures.^10–12^ Furthermore, next-generation sequencing (NGS) of large-scale samples has also uncovered novel rare/low-frequency variants in susceptible genes/loci for BMD, osteoporosis and fracture.^13,14^ Recently, the approach of Mendelian randomization was widely used to identify the causative risk factors for osteoporosis by using GWAS results.^15^

**Fig. 1:**
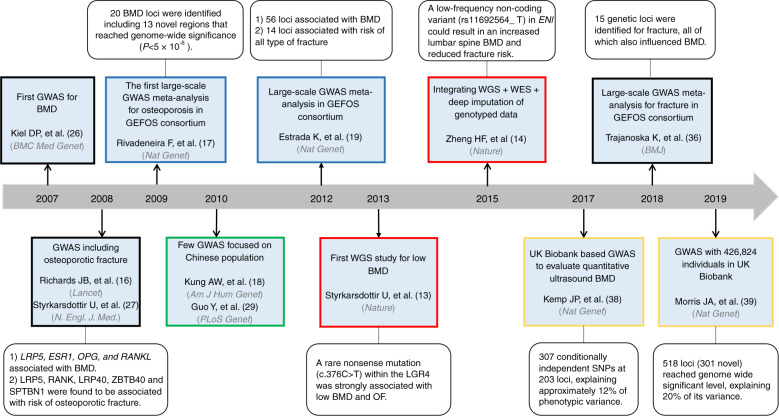
Timeline highlighting important milestones during the 12 years of GWAS discoveries for osteoporosis and related traits. Blue boxes indicate the studies from the GEFOS and GENOMOS consortia. The green box indicates the studies focused on the Chinese population. Red boxes indicate GWASs including rare variants. Yellow boxes indicate the UK Biobank-based GWAS. BMD bone mineral density, ESR1 estrogen receptor 1, GWAS genome-wide association study, LRP5 low-density lipoprotein receptor-related protein 5, LRP40 low-density lipoprotein receptor-related protein 4, OPG osteoprotegerin, RANK receptor activator of nuclear factor-kappa β, RANKL RANK ligand, SPTBN1 spectrin beta, nonerythrocytic 1, WES, whole-exome sequencing, WGS whole-genome sequencing, ZBTB40 zinc finger and BTB domain containing 40

In this article, we first reviewed the fruitful discovery achieved by GWASs and meta-analyses for osteoporosis and related traits in the last 12 years (Fig. 1). We introduced several newly raised conceptual models, such as omnigenic models and natural selection, which might explain the mystery of missing heritability of complex traits. We then summarized the clinical use of GWAS findings in the bone field, such as the identification of causal clinical risk factors, the development of drug targets, and disease prediction.

## GWAS in the bone field

### Measurement of bone mass

Most studies have focused on areal BMD (aBMD) obtained from a 2-dimensional projection scan with dual energy X-ray absorptiometry (DXA).^14,16–19^ The T-score is measured in standard deviation (SD), a mathematical term that calculates how much one’s bone mass varies from the average. It defines an individual’s bone mass as normal (above −1 SD), osteopenia (between −1.0 and −2.5 SD) and osteoporosis (below −2.5 SD).^20^ This measurement could be influenced by several different skeletal parameters, such as periosteal expansion, trabecular volumetric BMD (vBMD), cortical BMD, cortical thickness, trabecular number and trabecular thickness.^21^

Bone mass can also be assessed with other radiological imaging tools, such as quantitative computed tomography (QCT), which has the advantage of revealing unique bone information. Paternoster et al.^22^ performed the first GWAS on cortical vBMD measured by QCT and found that the genetic variant rs1021188 near the *RANKL* gene was associated with the density of cortical bone, and rs9287237 on *FMN2* was associated with the trabecular bone fraction, while the other three SNPs (rs271170 on *LINC00326* near *EYA4*, rs7839059 on *COLEC10* near *OPG* and rs6909279 on *CCDC170* near *ESR1*) had previously been reported to be associated with aBMD. However, QCT was not applicable to the WHO definition of osteoporosis that was based on DXA measurement, and QCT was more expensive with a higher dosage of exposure to radiation but might not predict fractures better than DXA measurement.^23^

An alternative method of estimating bone mass is derived from quantitative ultrasound (QUS). This measurement is quick, safe, and relatively inexpensive and can therefore be assessed in very large sample sizes, such as ~500 thousand samples in the UK Biobank. The advantages over DXA make QUS a complementary (not replacement) approach to bone health assessment. QUS consists of the use of two separate ultrasound measurements, speed of sound (SOS) and broadband ultrasound attenuation (BUA), typically at the heel. Measures of estimated BMD derived from ultrasound were moderately correlated with DXA-derived BMD at the hip and spine.^24^ A meta-analysis of GWASs^25^ using heel ultrasound parameters identified a novel locus (rs597319 near *TMEM135*) and replicated 6 previously reported loci (*ESR1*, *SPTBN1*, *RSPO3*, *WNT16*, *DKK1* and *GPATCH1*).

### Early GWAS design

**Fig. 2:**
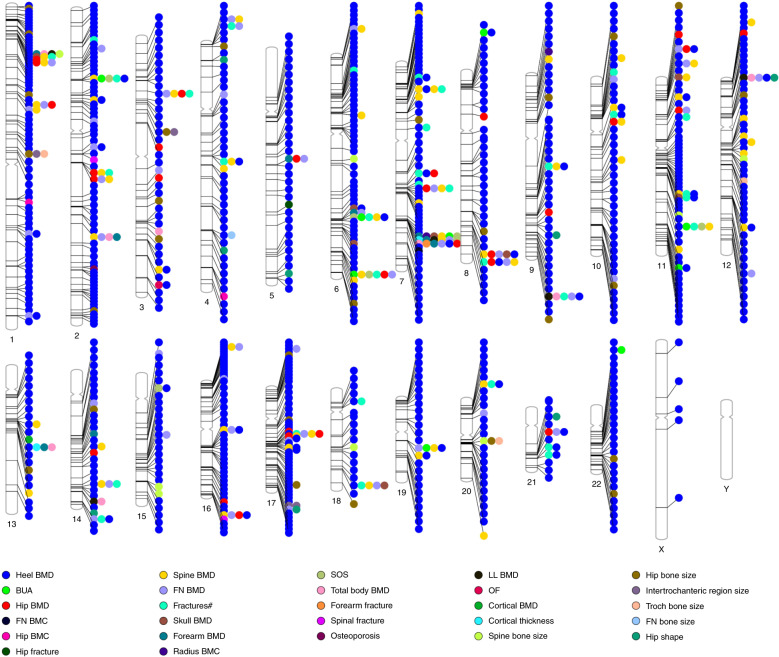
Genetic loci reported by the GWAS catalog for osteoporosis and related traits. ^#^ Fracture occurring at any site, except fingers, toes and skull, after age 18. BMC bone mineral content, BMD bone mineral density, BUA broadband ultrasound attenuation, FN femoral neck, LL lower limbs, OF osteoporotic fractures, SOS speed of sound

It has been established that the variation in BMD is the most important predictor for osteoporosis and fracture. Therefore, GWASs for osteoporosis mainly investigated the effect of genetic influence on BMD. In 2007, Kiel et al.^26^ published the first GWAS, including 1 141 Framingham Heart Study subjects, and they identified 40 SNPs that could potentially be associated with several bone phenotypes (Fig. 1). Unfortunately, owing to the small sample size, none of the *P* values exceeded the threshold of genome-wide significance (*P* < 5 × 10^−8^). In 2008, two GWASs were published and identified 4 loci associated with BMD (*LRP5*, *ESR1*, *OPG*, and *RANKL*); in addition, *LRP5*, *RANK*, *LRP40*, *ZBTB40*, and *SPTBN1* were found to be associated with the risk of osteoporotic fracture (Fig. 1).^16,27^ Soon afterwards, a deluge of GWASs were conducted on osteoporosis and related traits (Fig. 2, Table 1 and Supplemental Table 1).

**Table 1:** Genome-wide association studies conducted on osteoporosis and related traits

Studies	Traits	Discovery sample number and ancestry		Replication sample number and ancestry	Design	Population	Measurement
Kiel DP (2007)^26^	BMD (spine, femoral neck), hip geometry	1 117 European		–	GWAS	Adult	DXA
Richards JB (2008)^16^	BMD (spine, femoral neck), osteoporosis, osteoporotic fractures	2 094 European women		6 463 European	GWAS	91.8% women	DXA
Styrkarsdottir U (2008)^27^	BMD (spine, total hip or femoral neck), low-trauma fractures	5 861 European		7 925 European	GWAS	Elderly adults	DXA
Liu YZ (2008)^51^	Bone size (hip), low-trauma hip fractures	1 000 European		1 216 European; Chinese (266 cases, 177 controls)	GWAS	Adult	DXA
Styrkarsdottir U (2009)^144^	BMD (spine, hip), low-trauma fractures	6 865 European		8 510 European	GWAS	Adult	DXA
Xiong DH (2009)^145^	BMD (spine, total hip, femoral neck)	1 000 European		4 925 European, 3 655 Chinese, 908 African	GWAS	Adult	DXA
Timpson NJ (2009)^44^	BMD (femoral neck, total body minus the head)	1 518 European		4 312 European	GWAS	Children	DXA
Liu YZ (2009)^146^	BMD (spine, femoral neck)	1 000 European		3 355 European	GWAS	Adult	DXA
Rivadeneira F (2009)^17^	BMD (spine, femoral neck)	19 195 European		–	GWAS-meta	Adult	DXA
Guo Y (2010)^29^	Osteoporotic fractures (hip)	Asian Chinese (350 cases and 350 controls)		Asian Chinese (390 cases and 516 controls) BMD (9 962 European and Chinese)	GWAS	Elderly adults	DXA
Kung AW (2010)^18^	BMD (spine, femoral neck), osteoporotic fractures	785 Asian Chinese		13 913 European,1 584 Asian Chinese	GWAS	Adult	DXA
Koller DL (2010)^46^	BMD (spine, femoral neck)	1 524 European		669 European	GWAS	Premenopausal women	DXA
Guo Y (2010)^147^	BMD (femoral neck)	983 European		2 557 European	GWAS	Adult	DXA
Hsu YH (2010)^148^	BMD (spine, femoral neck), geometric indices of the hip	3 569 European		7 633 European women	GWAS-meta	Elderly adults	DXA
Tan L (2010)^149^	BMD (forearm)	1 000 European		1 628 East Asian	GWAS	Adult	DXA
Paternoster L (2010)^22^	Cortical BMD	1 934 European		3 835 European	GWAS-Meta	Adolescent, adult	pQCT
Kou I (2011)^30^	Osteoporosis	Japanese (713 cases and 3 094 controls)		Japanese (1 566 cases and 1 577 controls)	GWAS	Elderly adults	DXA
Duncan EL (2011)^150^	BMD (spine, total hip, femoral neck)	1 955 European		20 898 European	GWAS	Postmenopausal women	DXA
Lei SF (2012)^52^	Bone size (spine)	1 627 Chinses		1 728 European	GWAS	Adult	DXA
Estrada K (2012)^19^	BMD (spine, femoral neck), low-trauma fractures	32 961 European, East Asian		50 933 European	GWAS-meta	Elderly adults	DXA
Zheng HF (2012)^48^	BMD (forearm), cortical thickness, forearm fracture	5 672 European; Fracture: 2 023 cases, 3 740 controls, European			GWAS-Meta	Adult	DXA
Medina-Gomez, C (2012)^45^	BMD (total body minus the head)	2 600 European		11 052 European	GWAS	Children, adult	DXA
Liu CT (2012)^49^	BMD (spine, femoral neck)	25 353 European		24 763 European, East Asian and others	GWAS-Meta	Adult	DXA
Guo YF (2013)^53^	Bone size (hip)	1 627 Chinese		2 286 European	GWAS	Adult	DXA
Koller DL (2013)^47^	BMD (spine, femoral neck)	4 061 European, Asian		4 744 European, Asian	GWAS-meta	Premenopausal women	DXA
Deng FY (2013)^151^	Bone size (spine)	2 286 European		3 503 European; 1 627 Chinses	GWAS	Adult	DXA
Hwang JY (2013)^32^	Any low-trauma osteoporotic fractures	Korean (288 cases and 1 139 controls)		Asian (831 cases and 2 305 controls)	GWAS-meta	Elderly adults	Radiographs
Zheng HF (2013)^152^	BMD (forearm), forearm fracture	5 866 European, 715 Mexican American; forearm fracture: 2 023 cases and 3 740 controls			GWAS-Meta	Adult	DXA
Oei L (2014)^153^	Osteoporotic vertebral fractures	329 cases and 2 666 controls, European		26 217 European, 1 294 East Asian	GWAS	Elderly adults	Radiographs
Zhang L (2014)^35^	BMD (spine, total hip, femoral neck)	8 472 European, 1 547 East Asian, 1 124 others		10 732 European, 5 139 East Asian	GWAS-meta	Adult	DXA
Kemp JP (2014)^154^	BMD (forearm, lower limb, skull, total body minus the head)	8 007 European, 1 409 others		–	GWAS-Meta	Children	DXA
Chesi A (2015)^43^	BMD (forearm), BMC (forearm)	908 European; 163 others		481 European	GWAS	Children	DXA
Zheng HF (2015)^14^	BMD (spine, femoral neck, forearm), fractures#	BMD (total = 53 236) Fracture (total = 508 253)		–	WGS, GWAS-meta	Adults	DXA
Tan LJ (2015)^155^	BMD (spine, total hip)	826 Chinese		1 728 European;709 African American or Afro-Caribbean;408 Hispanic or Latin American	GWAS	Adult	DXA
Styrkarsdottir U (2016)^156^	BMD (spine, total hip or whole body), osteoporosis, osteoporotic fractures	European (2 894 cases, 206 485 controls)		–	WGS	Adult	DXA
Styrkarsdottir U (2016)^157^	BMD (spine, hip), osteoporotic fractures	BMD (20 100 Icelanders, 10 091 European and East Asian) osteoporotic fractures (10 389 cases and 264 522 controls)			GWAS	Adult	DXA
Mullin BH (2016)^158^	BMD (spine, total hip, femoral neck)	1 042 European		5 654 European	GWAS	Adult	DXA
Hwang JY (2016)^33^	SOS	1 895 Asian Korean		7 263 Asian Korean	GWAS-Meta	Adult	Ultrasound
Taylor KC (2016)^159^	Fractures#	10 305 African American or Afro-Caribbean		–	GWAS-meta	Only women	DXA, radiographs, self-report
Choi HJ (2016)^160^	BMD (spine, total hip, femoral neck)	2 729 Korean		1 547 Chinese; 3 237 European	GWAS	Adult	DXA
Pei YF (2016)^161^	BMD (hip Ward’s triangle)	4 305 European; 1 579 Asian; 1 295 others			GWAS-Meta	Adult	DXA
Pei YF (2016)^162^	BMD (hip trochanter and intertrochanter)	6 912 European		971 European, 1 291 others	GWAS	Adult	DXA
Chesi A (2017)^50^	BMD (spine, total hip, femoral neck, forearm)	933 European American		486 European American	GWAS	Children	DXA
Mullin BH (2017)^163^	SOS, BUA	1 268 European		1 610 and 13 749 European	GWAS-Meta	Adult	QUS
Villalobos-Comparán M (2017)^164^	BMD (spine, femoral neck) Bone geometry (femoral neck)	411 Hispanic or Latin American		420 Hispanic or Latin American	GWAS	Postmenopausal women	DXA
Kemp JP (2017)^38^	BMD (heel)	142 487 European		–	GWAS	Adult	QUS
Peng C (2017)^165^	BMD (spine, femoral neck)	53 236 NR; 5 152 East Asian		–	GWAS-meta	Adult	DXA
Lu S (2017)^166^	BMD (spine, hip and total body)	2 069 European		–	GWAS	Adult	DXA
Alonso N (2018)^167^	Osteoporotic vertebral fractures	European (1 553 cases, 4 340 controls)		European (1 028 cases, 3 762 controls)	GWAS	Only women	DXA
Inaba H (2018)^168^	BMD (spine)	235 European; 48 African American or Afro-Caribbean; 13 NR			GWAS	Children	QCT
Pei YF (2018)^169^	BMD (spine, femoral neck)	37 657 European; 1 539 East Asian; 1 295 others		–	GWAS-meta	Adult	DXA
Lin X(2018)^170^	BMD (femoral neck)	49 988 European		–	GWAS	Adult	DXA
Kim SK (2018)^171^	BMD (heel), osteoporosis and fracture	394 929 European; osteoporosis: 455 cases, 28 819 controls Fracture: 59 378 cases, 348 296 controls			GWAS	Adult	QUS
Qiu C (2018)^172^	BMD (spine)	1 547 Asian, 3 237 Caucasian, 712 African, 409 Hispanic	–		GWAS meta	Adult	DXA
Trajanoska K (2018)^36^	Fractures#	37 857 cases, 227 116 controls, European or East Asian	147 200 cases, 150 085 controls, European		GWAS-meta	Adult	Diagnosis, self-report
Gregson CL (2018)^173^	BMD (spine, total hip)	1 380 European	30 970 NR		GWAS	Adult	DXA
Naito T (2018)^31^	BMD (spine, femoral neck)	Japanese (173 cases, 81 controls)	–		GWAS	Adult	DXA
Liang X (2018)^174^	BMD (spine, hip, femoral neck, forearm, total body)	2 286 European; 3 404 Framingham Heart Study (FHS) subjects			GWAS	Adult, children	DXA
Morris JA (2019)^39^	BMD (heel), fracture#	426 824 individuals		–	GWAS-Meta	Adult	QUS, diagnosis, self-report
Hsu YH (2019)^175^	Hip geometry	18 719, mostly European		Mostly European (*N* > 9 000)	GWAS-Meta	Adult	DXA
Baird DA (2019)^54^	Hip shape	10 217 European, 5 717 NR		–	GWAS-Meta	Adult	DXA
Styrkarsdottir U (2019)^56^	Bone size (spine, hip, femoral neck, trochanter, intertrochanteric region)	European (*N* ≥ 28 954)		European and East Asian (*N* = 13 608–21 277)	GWAS	Adult	DXA
Zhang H (2020)^55^	BMD (hip) Bone size (hip)	3 267 European; 1 619 East Asian; 843 African American or Afro-Caribbean; 446 Hispanic or Latin American			GWAS	Adult	DXA

*BMD* bone mineral density, *BMC* bone mineral content, *BUA* broadband ultrasound attenuation, *DXA* dual-energy X-ray absorptiometry, *GWAS* genome-wide association study, *NA* not available, *QCT* quantitative computed tomography, *pQCT* peripheral QCT, *QUS* quantitative ultrasound, *SOS* speed of sound, *WGS* whole-genome sequencing; # fracture occurring at any site, except fingers, toes and skull, after age 18

### GWASs in the East Asian population

It is worth noting that the success of GWASs mainly came from studies performed in Caucasian populations, while only a few GWASs focused on East Asian populations (Fig. 1 and Table 1). Yang et al.^28^ performed a case-control GWAS in 700 elderly Chinese Han subjects (350 hip fracture patients and 350 healthy matched controls) and found that *UGT2B17* copy number variation was associated with hip fracture. The same GWAS dataset was reanalyzed by Guo et al.^29^ who found that the rs13182402 SNP in *ALDH7A1* at 5q31 was strongly associated with hip fracture (Fig. 1).^29^ Kung et al.^18^ conducted a GWAS and meta-analysis of BMD and fragility fractures in Chinese women (Hong Kong population) and found that the intronic SNP rs2273061 in the *JAG1* gene was strongly associated with the BMD of lumbar vertebrae (Fig. 1). The first GWAS of osteoporosis conducted in a Japanese population^30^ found that a common variant (rs7605378 on *FONG*) at 2q33.1 conferred the risk of osteoporosis in elderly individuals using a total of ~6 700 subjects.^30^ Recently, a GWAS with only 254 Japanese patients with inflammatory bowel disease (IBD) found that no SNPs reached genome-wide significance (*P* < 5 × 10^−8^) for femoral neck (FN) and lumbar spine (LS) BMD.^31^ Hwang et al.^32^ performed an association study in 1 119 fracture patients and 3 444 controls in Korean and Japanese populations and found a new *MECOM* locus associated with osteoporotic fracture [*P* = 3.59 × 10^−8^, odds ratio (OR) = 1.39]^32^. Another multistage GWAS meta-analysis identified a novel heel SOS locus (rs2446422 on *GLDN*) in the Korean population,^33^ and the allele-specific epigenetic modifications of the SNP were confirmed using ENCODE annotations.^33^

### GWAS meta-analysis

The allelic architectures of BMD and osteoporosis are likely to be multifactorial, with each factor imparting a relatively small effect. The identification of these loci with weak effects required studies with comprehensive coverage of the genome and very large sample sizes. The Genetic Factors of Osteoporosis (GEFOS) consortium (www.gefos.org) and the Genetic Markers for Osteoporosis (GENOMOS) consortium (www.genomos.eu) were employed to maximize the samples available for large GWAS meta-analyses, with a consequent increase in statistical power and new locus discovery. In 2009, a meta-analysis (GEFOS-1) of femoral neck and lumbar spine BMD was performed within the GEFOS consortium with 19 195 subjects of northern European descent and identified 20 BMD loci, including 13 novel regions that reached genome-wide significance (*P* < 5 × 10^−8^)^17^ (Fig. 1 and Table 1). In 2012, the GEFOS consortium released their second-round GWAS meta-analysis results (GEFOS-2);^19^ compared to the first GEFOS meta-analysis, the sample size of the study population increased significantly, which led to the identification of 56 loci associated with BMD (Fig. 1 and Table 1). However, not all genome-wide significant results of the first GEFOS meta-analysis could be replicated in the second study because increasing the sample size could also lead to sample heterogeneity.^34^ GEFOS-2 also revealed 6 loci associated with the risk of all types of fractures (*FAM210A*, *SLC25A13*, *LRP5*, *MEPE*, *SPTBN1, and DKK1*);^19^ however, the definition of fracture in this study was quite heterogeneous, including hip, spine, wrist and other types of fractures. Therefore, these findings should be interpreted with caution before independent validation in other samples with homogeneous fracture types.^34^ Zhang et al.^35^ conducted a three-stage GWAS meta-analysis, and two novel loci were identified in the pooled sample of males and females (*SMOC1*) and in the female-specific sample (*CLDN14*); they also independently confirmed 13 previously reported loci (*ZBTB40*, *GPR177*, *FGFRL1*, *MEPE*, *MEF2C*, *ESR1*, *SHFM1*, *WNT16*, *OPG*, *SOX6*, *LRP5*, *AKAP11*, and *FOXL1*). Further gene expression analysis in osteogenic cells implied a potential functional association of the *SMOC1* and *CLDN14* genes in bone metabolism^35^. For fracture risk, the largest GWAS meta-analysis to date, including 25 cohorts from Europe, the United States, East Asia and Australia, identified 15 genetic loci for fracture, all of which also influenced BMD.^36^

### GWASs including rare variants

Early GWAS design included only common variants [minor allele frequency (MAF) > = 5%] and poorly covered low-frequency (1% <=MAF < 5%) and rare variants (MAF < 1%). Next-generation sequencing technology provides an approach to capture rare and low-frequency variants, which might be identified to be associated with complex traits with large effects. Styrkarsdottir et al.^13^ performed the first whole-genome sequencing (WGS) study for BMD in an Icelandic population and found a rare nonsense mutation (c.376 C > T) within *LGR4* that was strongly associated with low BMD and osteoporotic fracture. However, the mutation was not present in the public Exome Variant Server (EVS) database or in the Australian samples (Fig. 1)^13^. In 2015, Zheng et al.^14^ integrated WGS data (*n* = 2 882), whole-exome sequencing data (*n* = 3 549), deep imputation of genotyped data (*n* = 26 534)^37^, and de novo replication genotyping data (*n* = 20 271) and found that a low-frequency noncoding variant (rs11692564_T, near *EN1*, MAF = 1.6%) could result in an increased lumbar spine BMD (effect size = +0.20 standard deviation, *P* = 2 × 10^−14^) and reduced fracture risk (OR = 0.85, *P* = 1 × 10^−11^) (Fig. 1). Conditional loss of *En1* in a cre/flox mouse model resulted in osteopenia and increased skull bone resorption via an indirect effect since *En1* was not expressed in osteoclasts.^14^

### Large-scale biobank based GWASs

The UK Biobank (www.ukbiobank.ac.uk) recruited 502 647 individuals aged between 37 and 76 years from all over the country in 2006–2010, and the heel bone quality of the participants was evaluated by quantitative ultrasound SOS and BUA. In 2017, Kemp et al.^38^ conducted a GWAS of 142 487 individuals from the UK Biobank using BMD as estimated by quantitative ultrasound of the heel. They demonstrated that 307 conditionally independent SNPs attained a genome-wide significance level at 203 loci, explaining ~12% of phenotypic variance (Fig. 1). Next, they investigated the underlying mechanism of these SNPs by four steps (including a. bioinformatic, functional genomic annotation and human osteoblast expression studies; b. gene function prediction; c. skeletal phenotyping of 120 knockout mice with deletions in genes adjacent to lead independent SNPs; d. the analysis of gene expression in mouse osteoblasts, osteocytes and osteoclasts) and suggested that the *GPC6* gene was a novel determinant of BMD and the pathophysiology of osteoporosis.^38^

A new study by Morris et al. evaluated genetic determinants of BMD as estimated by heel quantitative ultrasound in 426 824 individuals, identifying 518 loci (301 novel) that reached a genome-wide significance level, explaining 20% of its variance (Fig. 1).^39^ They also undertook a meta-analysis of ~1.2 million individuals and identified 13 fracture loci (all associated with heel BMD), highlighting the importance of BMD as a determinant of fracture risk.^39^ They found that target genes were enriched in those known to influence bone density and strength from cell-specific features (maximum OR = 58, *P* = 1 × 10^−75^) and found an increased abnormal skeletal phenotype frequency through the phenotyping of 126 knockout mice with disruptions in predicted target genes.^39^ Finally, *DAAM2* showed critical effects on bone strength, porosity, composition and mineralization.

While most BMD GWASs analyzed data derived from DXA, these two studies of UK Biobank data used estimated BMDs derived from calcaneus ultrasound. Though Pearson’s correlation coefficients between DXA and QUS parameters showed a moderate association (*r* = 0.42–0.61)^24,40^ and quantitative ultrasound had the ability to predict the occurrence of fractures in older women^41^ and men,^42^ there were some essential differences. The GWASs did not replicate 18 known loci from previous studies utilizing DXA-derived BMD measures, and 6 loci had opposite effects on heel BMD in the study of Kemp et al.^38^ compared to previous DXA BMD studies. Although these differences may be due to various reasons, differences in measurement by QUS and DXA were likely to be causes.

### Age- and sex-specific BMD GWASs

Although most studies have focused on adults, GWASs have also performed in younger individuals, including children,^43–45^ teenagers,^22^ and premenopausal women.^46,47^ The first GWAS reported for BMD in children identified the *SP7* locus, which encodes the transcription factor osterix, as being associated with whole-body BMD, and replication was subsequently achieved in adult lumbar spine BMD.^44^ Recently, Chesi et al.^43^ found that two loci achieved genome-wide significance: rs7797976 within *CPED1* in girls and rs7035284 near *MTAP* in boys at the distal radius. Actually, signals at the *CPED1-WNT16-FAM3C* locus have been previously reported to be associated with BMD at other skeletal sites in adults^17^ and children (skull and total body aBMD) of European ancestry^45^. Interestingly, this locus was also associated with cortical bone thickness, bone strength, and the risk of forearm fracture in adults;^48^ peak bone mass in premenopausal women;^47^ and BMD and fracture in elderly individuals.^19^ The integration of functional studies in *Wnt16* knockout mice revealed reductions in bone mineral content (BMC), bone area and bone strength.^45,48^ Both natural variation in humans and functional studies in *Wnt16* knockout mice demonstrated that *WNT16* was an important determinant of the bone mass at different body sites in children and adults and the risk of fracture, suggesting that this genetic effect acted over the whole lifetime.

To detect genetic variants influencing variability in peak BMD in premenopausal women, Koller et al. conducted a GWAS in 1 524 US Caucasian women (aged 20–45 years) and 669 African American women (aged 20–44 years). A novel gene, *CATSPERB*, was identified to be significant in femoral neck BMD.^46^ *CATSPERB* was not found to be significant in the meta-analyses of GEFOS-2, although the samples from the above study were included in GEFOS-2.^19^ Later, a meta-analysis was carried out restricting samples to premenopausal white women from 4 cohorts (*n* = 4 061, aged 20–45 years), and two loci (*WNT16*) and (*ESR1/C6orf97*) were identified to influence the peak bone mass at the lumbar spine and femoral neck.^47^ Only 4 out of the 56 GEFOS-2 loci^19^ were observed to have *P* values below 5 × 10^−5^ in this meta-analysis.

Although most of the published GWASs on skeletal phenotypes did not have adequate power to test sex-specific genetic effects,^49^ there was suggestive evidence for an interaction between sex and SNP rs1021188 (near *RANKL*) (*P* = 0.01), with a stronger association in males than females (at age 15, males −6.77 mg·cm^3^ per C allele, *P* = 2 × 10^−6^; females −2.79 mg·cm^3^ per C allele, *P* = 0.004).^22^ In the GEFOS-2 study,^19^ two loci (Xp22.31 in men and 8q13.3 in women) were discovered in the sex-stratified meta-analysis; however, only the locus in Xp22.31 (near *FAM9B*) showed significant heterogeneity (*P*_het_ = 1.62 × 10^−8^), and the imbalance in sample size between women and men and the conservative heterogeneity test limited the ability to identify sex-specific findings. In a study of European American children (*n* = 1 419),^50^ four novel loci (*IZUMO3*, *RBFOX1*, *SPBT*, and *TBPL2*) were identified to be associated with BMD at the 1/3 distal radius, spine, total hip and femoral neck, two of which were sex-specific loci (*SPTB* in females and *IZUMO3* in males).

### Bone size/geometry GWASs

Bone size (BS) is also an important factor that influences bone geometry and bone strength. To date, a limited number of GWASs for BS have been conducted compared to studies on BMD. In Table 1, we summarized the current GWASs and meta-analyses in bone size/geometry. In early studies, GWASs of the bone area of the hip or lumbar spine using DXA did not find significant loci,^26,51–53^ possibly due to the small sample size. Recently, a study of a hip shape model (HSM) derived from statistical shape modeling of DXA scans found 8 loci associated with hip shape,^54^ and another GWAS meta-analysis identified 22 significant loci (*P* < 5.0 × 10^−8^) for hip bone size.^55^ Styrkarsdottir et al. reported a large GWAS of bone size using a simple parameter from DXA scans, the bone area,^56^ they found that 8 loci for the lumbar spine area, 5 loci for the total hip area, 4 loci for the intertrochanteric area, 3 loci for the trochanter area, and 1 locus for the femoral neck area satisfied the criteria of genome-wide significance (Table 1).

## GWAS findings cannot perfectly explain the variance in bone mass

GWASs on osteoporosis and related traits have made great achievements in the past 12 years and have highlighted many genes/loci and related biological pathways that contribute to the pathophysiology of osteoporosis and/or fracture, such as the RANK-RANKL-OPG and WNT signaling pathways. These pathways are functionally relevant to bone metabolism and endochondral ossification, and their contribution to osteoporosis has been well established.^57^ However, at the same time, similar to other complex traits, the variance in bone mass could not be fully explained by GWAS findings.

### Missing heritability and beyond

The genetic architecture of osteoporosis and fracture involves both common and rare functional variants,^58,59^ and the effect sizes of low-frequency and rare variants by genetic burden are larger than those of common variants.^14^ BMD is also a highly heritable trait, and the genetic effect was estimated to account for as much as 75% of the variance in BMD at the site of the femoral neck;^60^ however, only a small portion of heritability was explained by loci identified by GWASs. For example, in the GEFOS-1 study,^17^ 15 LS-SNPs combined explained ~2.9% of the variance in LS-BMD, and 10 FN-SNPs combined explained ~1.9% of the variance in FN-BMD with 19 195 subjects. In the GEFOS-2 study,^19^ 63 SNPs explained 5.8% of the total genetic variance in FN-BMD among ~84 000 individuals. To date, the number of associated loci has increased to ~1 000, explaining 20% of the variance in eBMD among 426 824 subjects.^39^ GWASs rely on the “common disease-common variant” hypothesis and lead to the identification of multiple genetic variants that explain, in aggregate, only a small portion of the BMD variance. This has been referred to as the mystery of the “missing heritability”.^61^ Therefore, larger-scale, better-powered GWASs could identify more variants, but it seems that the variance explained by these common variants is likely to remain minor.

Unlike Mendelian diseases that are caused by mutations in coding regions, most of the associated SNPs for osteoporosis and related traits are found in noncoding intergenic and intronic regulatory regions.^62^ Therefore, the greatest challenge was to understand the functional consequences of these SNPs and to accurately elucidate the biological mechanism by which these genes and SNPs act. To date, only a small fraction of SNPs/genes and their functional mechanisms have been successfully characterized,^63^ and these variants or regions could be transcription factor binding sites that regulate or affect gene expression.^62^

### Polygenicity and negative selection

GWASs of osteoporosis and related traits often identified a number of SNPs that had significant p values but showed very low disease odds ratios (ORs). For example, in the GEFOS-2 study,^19^ 13 of 14 SNPs associated with any low-trauma osteoporotic fracture had ORs <1.10. A recent GWAS involving 53 184 fractures and 373 611 controls^39^ identified 14 association SNPs, all of which had ORs <1.10. In a GWAS of Chinese fractures, the highest OR of rs13182402 in the *ALDH7A1* gene was 2.25.^29^ Despite their statistical significance, the ORs were small and explained little about the genetic contribution to fracture.

Over the last few years, a commonly accepted explanation for the small OR was that osteoporosis was caused by a large number of interacting genes, each with a small effect size and additive increment to disease risk, called “polygenic inheritance”. It is known that common diseases have a polygenic genetic architecture.^64^ Thus, perhaps in many cases, the so-called problem of missing heritability might be synonymous with high polygenicity (defined as the **total number** of genetic loci or alleles with nonzero effects contributing to a phenotype).^61,65^ The classic polygenic model consists of contributions to disease risk from both common and rare variants.^61^ In 2018, using UK Biobank data, Zeng et al. confirmed that negative selection played a predominant role in shaping the relationship between effect size and MAF for complex traits.^66^ They found that 23 out of the 28 studied complex traits (including heel BMD) showed significant signatures of natural selection, and the genetic variants associated with heel BMD were under negative selection, with a moderate estimate (Sˆ = −0.381), where Sˆ reflected the strength of selection on the trait-associated SNPs.^66^

More recently, O’Connor et al. redefined polygenicity as the **effective number** of independently associated SNPs (Me). For the 33 complex traits they studied, the “Me” estimates for common SNPs ranged from 500 to 20 000, with a ‘Me’ estimate for heel BMD of ~800.^67^ This implied that most common SNPs were associated with complex traits and that heritability was spread evenly across the genome.^67^ They found that functionally important regions in the genome had higher polygenicity and higher heritability, but low-frequency SNPs had lower polygenicity than common SNPs on average. The conclusion was that negative selection not only constrained the effect sizes of common variants on average but also flattened their distribution across the genome.^67^

### Polygenicity and omnigenicity

Recently, Boyle et al.^68^ proposed the “omnigenic model” in which gene regulatory networks were fully interconnected; that is, all genes expressed in disease-related cells were considered to affect disease phenotype, but most heritability could be explained by effects on genes outside core pathways. This model tried to answer 2 questions: (1). Why do the lead hits from GWASs for any given trait contribute so little to heritability? (2). Why does so much of the genome contribute to heritability? The key feature of this model was the classification of genes as “core” (direct roles in disease) or “peripheral” (essentially all other expressed genes can transregulate core genes). In fact, the “omnigenicity model” is one scenario of the “polygenicity model”, in which the “polygenicity” is partitioned into different parts.

In the latest point of view, they defined the “core gene” as the only gene from which the gene product (protein or RNA for a noncoding gene) had a direct effect—not mediated through the regulation of another gene—on cellular and organismal processes, leading to a change in the expected value of a particular phenotype; “peripheral genes” were defined as those expressed in relevant cell types that could affect the phenotype only indirectly through regulatory effects on core genes.^69^ This model assumed that the relationship between each core gene and the expected phenotype value was a linear function of the gene expression level; moreover, each core gene was likely affected by large numbers of weak trans (peripheral) variants, and most trait heritability was mediated through trans effects.^69^

Based on this model, most variants that contributed to heritability tended to be spread across the whole genome, and genes with specific functions for osteoporosis or related traits could only explain little heritability. This **might** explain why some loci/genes identified by GWASs for BMD were considered to have no contribution to the pathophysiology of osteoporosis and/or fracture, while some genes that had known functional relevance to bone metabolism and endochondral ossification tended to be core genes. For example, *LRP5*, which encodes low-density lipoprotein receptor-related protein 5, could function as a coreceptor together with the seven-transmembrane-spanning Frizzled for Wnt proteins to regulate intracellular signal transduction by β-catenin,^70,71^ and the activation of the Wnt pathway results in cytoplasmic β-catenin accumulation. Consequently, β-catenin translocates to the nucleus and in turn regulates osteoblast proliferation and differentiation, thus determining bone mass.^72^ Osteoblasts produce RANKL following the binding of RANKL to RANK on the surface of osteoclastic precursors, and subsequently, NF-κB is activated and translocates into the nucleus and interacts with NFATc1 to trigger osteoclastogenic gene transcription.^73^ *OPG*, a member of the tumor necrosis factor (TNF) receptor superfamily (TNFRS), also known as TNFRS member 11B (*TNFRS11B*), can bind to *RANKL* to prevent its coupling with *RANK* and inhibit the maturation of osteoclasts as a result of reducing bone resorption. Notably, these genes related to the bone metabolism pathway, such as *LRP5*,^16^ *RANKL*,^22^ *ESR1*,^25,38,39^ *BMP4* (bone morphogenetic protein 4),^38,74^ and *WNT16*, were identified in GWAS signals.^38,45,48^

## Clinical relevance of GWAS findings

The ultimate goal of genetic study is to translate the discoveries into clinical practice. GWAS discoveries for osteoporosis and related traits in the past 12 years are undoubtedly more fruitful than previous linkage analyses and candidate gene association analyses, and hundreds of loci (thousands of SNPs) have been identified that are significantly and robustly associated with osteoporosis and related traits (Fig. 2, Table 1 and Supplemental Table 1). However, it is still too early to understand the function of novel proteins identified by GWASs. This review is not meant to describe novel discovered loci and their interactions. We assumed that there are three ways in which GWAS findings could provide important clinical insight for osteoporosis. First, GWAS results could be employed to investigate the causal risk factors for osteoporosis by using the Mendelian randomization approach. Second, new drug targets and anti-osteoporotic therapeutics should be investigated. Despite the small effect of common variants identified by GWASs, it should be noted that the effect size of the genetic variant on molecular phenotypes could be large, and the drug effect on targets could also be magnified (e.g., statins).^75^ Third, genetic information could be applied to “personalized” medicine, for example, disease prediction and risk stratification, leading to the overall improvement in disease prevention or intervention.

### Mendelian randomization approach to link clinical risk factors to osteoporosis and fracture

The identification of causative risk factors is essential for the prevention and treatment of osteoporosis, and a better understanding of causality could be conducive to further prevention strategies and clinical trials and to providing targets for effective lifestyle and drug intervention.^15,76^ Observational studies have identified associations of potential risk factors (for example, smoking, low body mass index (BMI), low vitamin D level, earlier age at menopause and physical inactivity) with fracture risk. However, because of confounding factors and reverse causality, bias might be introduced into observational studies, thereby reducing their reliability. The gold standard for evidence for causal effects could come from well-conducted randomized control trials (RCTs), but RCTs are resource-intensive and examine mainly short-term exposures. In addition, not all risk factors can be investigated by RCTs.^15^

**Fig. 3:**
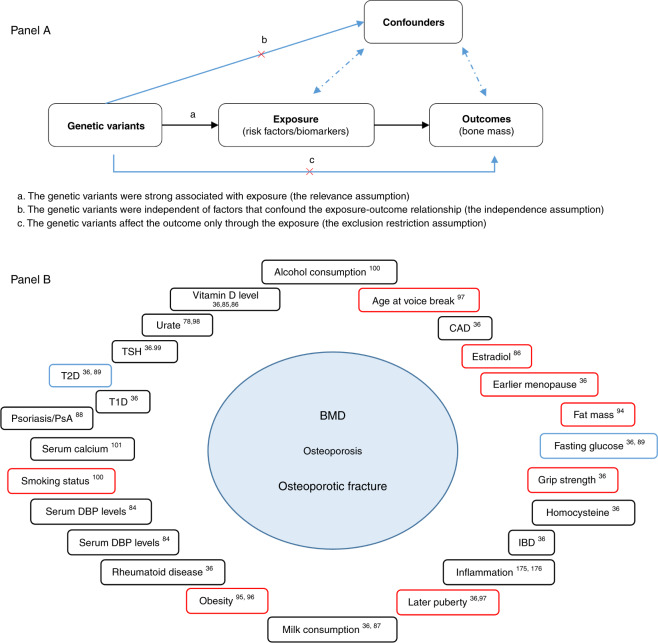
Mendelian randomization in bone field. **Panel A**: Principal of Mendelian randomization. **Panel B**: The causality between the clinical risk factors and osteoporosis from the current literature. Red boxes indicate the causal relationship. Black boxes indicate the noncausal relationship. Blue boxes indicate controversial results. BMD bone mineral density, CAD coronary artery disease, IBD inflammatory bowel disease, DBP vitamin D binding protein, PsA psoriatic arthritis, T1D type 1 diabetes, T2D type 2 diabetes, TSH thyroid stimulating hormone

Recently, Mendelian randomization (MR) analyses have been widely used to illustrate the causal effect between exposures and outcomes using large-scale GWAS summary statistic data.^77–79^ MR is a type of analytical approach that takes genetic variants associated with a risk factor (e.g., calcium) as instrumental variables (IVs) to examine the causality between exposure and outcome (e.g., BMD).^80,81^ Since the genetic alleles are randomly assorted during conception, MR analyses are less susceptible to confounding factors; additionally, MR analyses are robust to reverse causation bias because genotypes are unlikely to be affected by disease. Further information can be found in Supplemental Note Box 2. Three main assumptions must be applied when conducting Mendelian randomization analyses.^82^ First, the genetic variants should be strongly associated with exposure (the relevance assumption); second, the genetic variants should be independent of factors that confounded the exposure-outcome relationship (the independence assumption); and third, the genetic variants affect the outcome only through the exposure (the exclusion restriction assumption) (Fig. 3, Panel A). This approach has advantages over traditional observational studies by minimizing confounding bias. To date, the MR approach in the bone field has been applied predominantly to assess causal relationships between different factors and BMD, osteoporosis and fracture (Fig. 3, Panel B and Table 2). Among these factors, vitamin D level, inflammatory disease, obesity and diabetes were frequently investigated.

**Table 2:** Mendelian randomization studies in the bone field

Studies	Exposure	Outcome	IVs	Sample size; ethnicity	MR method	Unit	*P* value	Estimate (95% CI)	Interpretation
Timpson NJ (2009)^94^	Obesity (fat mass)	BMC	2 SNPs (fat mass)	Obesity (8 480) BMC (7 470), children; European	IV regression with 2SLS	1 g change in BMC per 1 kg change in fat mass	0.000 2	TB-BMC, 0.02 (−0.20, 0.15)	Fat mass is in the causal pathway for bone mass in children.
							0.03	UL-BMC, 0.46 (0.31, 0.61)	
							0.002	LL-BMC, 0.55 (0.41, 0.68)	
							2.30E-06	LS-BMC, 0.48 (0.33, 0.63)	
Warodomwichit D (2013)^96^	Obesity	BMD	1 SNP	Obesity (2 154) BMD (2 154), adults; Thai	IV regression with 2SLS	1 g.cm^−2^ change in BMD per 1 kg.m^−2^ change in BMI	0.01	TH-BMD, 0.02 (0.00, 0.03)	Obesity might be causally related to BMD at the femur but not at the spine.
							0.014	FN-BMD, 0.01 (0.00, 0.03)	
							NS	LS-BMD, 0.00 (−0.01, 0.01)	
Oeil L (2014)^176^	Inflammation	Fracture	29 SNPs (CRP)	Inflammation (6 386) Fracture (1 561), adults; American	Weighted genetic risk score	OR for fracture per 1 SD increase in CRP	0.23	Fracture, 1.00 (0.99, 1.00)	No causal association between CRP level and fracture.
Leong A (2014)^84^	Serum DBP levels	BMD	1 SNP	Serum DBP levels (2 254) BMD (2 254), adults; Canadian	IV regression with 2SLS	1 g.cm^−2^ change in BMD per 1 SD change in DBP	0.43	FN-BMD, −0.005 (−0.02, 0.01)	No causal association between DBP level and BMD.
Dalbeth N (2015)^98^	Urate	BMD	5 SNPs	Urate (2 501) BMD (2 501), adults; European	IV regression with 2SLS	1 g.cm^−2^ change in BMD per 1 mmol.L^−1^ change in urea levels	0.06	TF-BMD, −0.29 (−0.60, 0.01)	No causal association between urate and BMD.
							0.08	FN-BMD, −0.27 (−0.58, 0.03)	
							0.68	LS-BMD, 0.08 (−0.32, 0.48)	
Xiong A (2016)^78^	Urate	BMD	18 SNPs	Urate (1 322) BMD (1 322), adults; Chinese	IV regression with 2SLS	1 g.cm^−2^ change in BMD per 1 mmol.L^−1^ change in urea levels	0.5	TH-BMD, 0.19 (−0.36, 0.74)	No causal association between urate and BMD.
							0.53	FN-BMD, −0.19 (−0.42, 0.81)	
							0.26	LS-BMD, 0.39 (−0.26, 0.98)	
Kemp JP (2016)^95^	Obesity	BMD	32 SNPs (BMI)	Obesity (5 221) BMD (5 221), children; European	MR Egger; Multivariable MR	SD change in BMD per SD increase in BMI	0.78	SK-BMD, −0.02 (−0.20, 0.15)	Obesity is causally related to increase in BMD at all sites except the skull.
							<0.001	UL-BMD, 0.46 (0.31, 0.61)	
							<0.001	LL-BMD, 0.55 (0.41, 0.68)	
							<0.001	LS-BMD, 0.48 (0.33, 0.63)	
							<0.001	PE-BMD, 0.39 (0.34, 0.64)	
Li SS (2016)^85^	Vitamin D level	BMD	4 SNPs	Vitamin D level (1 824) BMD (1 824), postmenopausal women; Chinese	IV regression with 2SLS	1 g.cm^−2^ change in BMD per 1 log ng.mL^−1^ change in total 25OHD	0.326	TH-BMD, −0.04 (−0.13, 0.04)	No causal association between vitamin D and BMD.
							0.261	FN-BMD, −0.04 (−0.13, 0.03)	
							0.384	LS-BMD, 0.05 (−0.16, 0.06)	
Ahmad OS (2017)^89^	T2D	BMD	32 SNPs	T2D (149 821) BMD (32 961), adults; European	IVW approach	SD change in BMD per odds in log-odds of T2D	0.044	FN-BMD, 0.034 (0.001, 0.067)	Genetically increased T2D risk and genetically increased fasting glucose have weak positive effects on FN-BMD.
							0.148	LS-BMD, 0.026 (−0.01, 0.061)	
	Fasting glucose (FG)	BMD	30 SNPs	FG (133 010) BMD (32 961), adults; European	IVW approach	SD change in BMD per 1 mmol.L^−1^ increase in GF	0.034	FN-BMD, 0.13 (0.01, 0.25)	
							0.211	LS-BMD, 0.082 (−0.045, 0.21)	
	2-h glucose	BMD	6 SNPs	2hGlu (133 010) BMD (32 961), adults; European	IVW approach	SD change in BMD per 1 mmol.L^−1^ increase in 2hGlu	0.134	FN-BMD, 0.089 (−0.027, 0.20)	
							0.354	LS-BMD, 0.06 (−0.06, 0.18)	
Yang Q (2017)^87^	Milk consumption	BMD	1 SNP (lactose intolerance)	Milk consumption (32 961) BMD (32 961), adults; European	IVW approach	1 SD change in BMD per 1 SD change of milk consumption	NA	FA-BMD, 0.049 (−0.128, 0.226)	No causal association between adult milk intake and BMD.
							NA	FN-BMD, −0.015 (−0.089, 0.059)	
							NA	LS-BMD, 0.015 (−0.073, 0.104)	
Huang JV (2017)^177^	Inflammation	BMD	16 SNPs (CRP)	BMD (32 961); European	MR-Egger IVW approach	1 g.cm^−2^ change in BMD per 1 log mg.L^−1^ change in total hsCRP	0.506	FA-BMD, 0.054 (NA)	No causal association between hsCRP and BMD
							0.726	FN-BMD, −0.014 (NA)	
							0.184	LS-BMD, −0.074 (NA)	
Cousminer DL (2018)^97^	Later puberty	BMD	331 SNPs	733 girls; European	Two-sample MR	1 SD change in BMD per 1 year later onset of puberty	0.004 6	LS-BMD −0.179	A causal association between later puberty and LS-BMD.
	Age at voice break	BMD	43 SNPs	685 boys; European	Two-sample MR	1 SD change in BMD per 1 year earlier onset of age at voice break	0.000 3; 7.04E-05	LS-BMD −0.119; FN-BMD: −0.113	A causal association between later puberty and LS/FN-BMD.
Larsson SC (2018)^86^	Estradiol	BMD	1 SNP	Estradiol (2 767) eBMD (32 965), adults; European	IVW approach	1 SD change in BMD and g.cm^−2^ in eBMD per 10% increase in estradiol	4.60E-06	FN-BMD, 0.038 (NA)	A causal association between serum estradiol levels and increase BMD.
							0.001	LS-BMD, 0.031 (NA)	
							6.00E-18	eBMD, 0.030 (NA)	
	Vitamin D level	BMD	5 SNPs	Vitamin D (42 274) BMD (32 961) eBMD (142 487), adults; European	IVW approach	1 SD change in BMD per 1 SD change in 25OHD (g.cm^−2^ eBMD)	0.37	FN-BMD, 0.02 (−0.03, 0.07)	No causal association between vitamin D and BMD.
							0.49	LS-BMD, 0.02 (−0.04, 0.08)	
							0.02	eBMD, -0.03 (−0.05, −0.01)	
van Vliet NA (2018)^99^	TSH	BMD	20 SNPs	TSH (26 420) BMD (32 735), adults; European (mostly)	Two sample MR IVW approach	SD change in BMD per 1 SD decrease in serum TSH level	0.92	FN-BMD: 0.003 (−0.053, 0.048)	No causal association between serum TSH levels and BMD.
							0.73	LS-BMD: 0.010 (−0.069, 0.049)	
Guo R (2018)^100^	Smoking status	BMD	139-142 SNPs	Smoking status (32 735) BMD (445 921), adults; European (mostly)	Two-sample MR IVW approach	NA	0.053	FN-BMD: −0.139 (NA)	A causal association between smoking and decreased heel BMD.
							0.976	LS-BMD: −0.003 (NA)	
							0.077	FA-BMD: −0.264 (NA)	
							0.003	Heel BMD: −0.053 (NA)	
	Alcohol consumption	BMD	5–6 SNPs	Alcohol consumption (32 735) BMD (445 921), adults; European (mostly)	Two-sample MR IVW approach	NA	0.964	FN-BMD: −0.008 (NA)	No causal association between alcohol consumption and BMD.
							0.742	LS-BMD: 0.067 (NA)	
							0.593	FA-BMD: 0.194 (NA)	
							0.822	Heel BMD: 0.010 (NA)	
Trajanoska K (2018)^36^	T2D	Fracture	38 SNPs	T2D: 56 862 (12 171 cases) Fracture: 185 057 cases, 377 201 controls; mostly European	Two sample MR; MR-Egger	OR of fracture per doubling in odds of T2D susceptibility	0.37	Fracture: 0.99 (0.99, 1.01)	No causal association between T2D and fracture risk.
	T1D	Fracture	19 SNPs	T1D: 26 890 (9 934 cases) Fracture: 185 057 cases, 377 201 controls	Two sample MR; MR-Egger	OR of fracture per doubling in odds of T1D susceptibility	0.57	Fracture: 1.00 (1.00, 1.01)	No causal association between T1D and fracture risk.
	Fasting glucose	Fracture	35 SNPs	Fasting glucose (58 074) Fracture: 185 057 cases, 377 201 controls	Two sample MR; MR-Egger	OR of fracture per 1 SD increase in fasting glucose level	0.24	Fracture: 1.04 (0.97, 1.12)	No causal association of fasting glucose levels with fracture risk.
	CAD	Fracture	38 SNPs	CAD: 107 432 (41 513 cases) Fracture: 185 057 cases, 377 201 controls	Two sample MR; MR-Egger	OR of fracture per doubling in odds of CAD susceptibility	0.76	Fracture: 1.00 (0.99, 1.02)	No causal association between CAD and fracture risk.
	Rheumatoid disease	Fracture	30 SNPs	Rheumatoid disease: 58 284 (14 361 cases) Fracture: 185 057 cases, 377 201 controls; mostly European	Two sample MR; MR-Egger	OR of fracture per doubling in odds of rheumatoid disease susceptibility	0.14	Fracture: 1.01 (1.10, 1.02)	No causal association between rheumatoid disease and fracture risk.
	Vitamin D	Fracture	4 SNPs	Vitamin D: 33 996 Fracture: 185 057 cases, 377 201 controls; mostly European	Two sample MR MR-Egger	OR of fracture per 1 SD decrease in 25OHD	0.07	Fracture: 0.84 (0.70, 1.02)	No causal association of decreased 25OHD levels with increased fracture risk.
	Dairy calcium intake	Fracture	1 SNP (lactose intolerance)	Dairy calcium intake: 171 213 Fracture: 185 057 cases, 377 201 controls; mostly European	Two sample MR MR-Egger	OR of fracture per 1 SD increase in milk consumption	0.94	Fracture: 1.01 (0.80, 1.23)	No causal association between milk consumption and fracture risk.
	FN-BMD	Fracture	43 SNPs	FN-BMD: 32 961 Fracture: 185 057 cases, 377 201 controls; mostly European	Two sample MR MR-Egger	OR of fracture per 1 SD decrease in FN-BMD	<0.001	Fracture: 1.55 (1.48, 1.63)	A causal association between decreased FN-BMD and increased fracture risk.
	LS-BMD	Fracture	40 SNPs	LS-BMD: 31 800 Fracture: 185 057 cases, 377 201 controls; mostly European	Two sample MR MR-Egger	OR of fracture per 1 SD decrease in LS-BMD	<0.001	Fracture: 1.43 (1.37, 1.50)	A causal association between decreased LS-BMD and increased fracture risk.
	Homocysteine	Fracture	13 SNPs	Homocysteine: 44 147 Fracture: 185 057 cases, 377 201 controls; mostly European	Two sample MR MR-Egger	OR of fracture per 1 SD increase in homocysteine level	0.78	Fracture: 0.98 (0.92, 1.05)	No causal association between homocysteine level and fracture risk.
	Inflammatory bowel disease (IBD)	Fracture	151 SNPs	IBD: 34 652 (12 882 cases) Fracture: 185 057 cases, 377 201 controls; mostly European	Two sample MR MR-Egger	OR of fracture per doubling in odds of inflammatory bowel disease susceptibility	0.92	Fracture: 1.00 (1.10, 1.01)	No causal association between inflammatory bowel disease and fracture risk.
	TSH	Fracture	20 SNPs	TSH: 26 523 Fracture: 185 057 cases, 377 201 controls; mostly European	Two sample MR MR-Egger	OR of fracture per 1 SD decrease in serum TSH level	0.78	Fracture: 0.99 (0.94, 1.04)	No causal association between serum TSH levels and fracture risk.
	Grip strength	Fracture	15 SNPs	Grip strength: 142 035 Fracture: 185 057 cases, 377 201 controls; mostly European	Two sample MR MR-Egger	OR of fracture per 1 SD increase in grip strength	0.01	Fracture: 2.14 (1.13, 4.04)	A causal association between decreased grip strength and fracture risk.
	Age of puberty	Fracture	54 SNPs	Age of puberty: 182 416 Fracture: 185 057 cases, 377 201 controls; mostly European	Two sample MR MR-Egger	OR of fracture per 1 SD change, i.e., 3.9 years earlier menopause	0.05	Fracture: 1.10 (1.00, 1.21)	No causal association between earlier menopause and fracture risk.
	Age at menopause	Fracture	106 SNPs	Age at menopause: 69 360 Fracture: 185 057 cases; 377 201 controls; mostly European	Two sample MR MR-Egger	OR of fracture per 1 SD change, i.e., 1.4 years late puberty	0.04	Fracture: 1.06 (1.00, 1.13)	A causal association between late puberty and increased fracture risk.
Cerani A (2019)^101^	Serum calcium	BMD	1 SNP	Serum calcium: 61 079 BMD: 426 824; mostly European	IVW approach	1 SD change in BMD per 1 SD change in serum calcium concentration	0.85	heel BMD, 0.003 (−0.059–0.066)	No causal association between serum calcium consumption and heel BMD.
	Serum calcium	Fracture	1 SNP	Serum calcium: 61 079 Fracture: 76 549 cases, 470 164 controls; mostly European	IVW approach	OR of fracture per 1 SD increase in serum calcium concentration	0.85	Fracture, 1.01 (0.89–1.15)	No causal association between serum calcium consumption and fracture risk.
Xia (2020)^88^	Psoriasis	eBMD	60 SNPs	301 667, European	One-sample MR	SD change in BMD per odds in log-odds of psoriasis susceptibility	0.24	heel BMD, − 0.04 (−0.11–0.029)	No causal association between psoriasis and heel BMD.
				Psoriasis: 19 032 cases, 286 769 controls eBMD: 462 824; European	Two sample MR		0.28	heel BMD, −0.002 (−0.009–0.002)	
	Psoriasis	Fracture	60 SNPs	Psoriasis: 19 032 cases, 286 769 controls Fracture: 45 087 cases, 317 775 controls; European	Two sample MR	OR of fracture per doubling in odds of psoriasis susceptibility	0.72	Fracture, 1.00 (0.99–1.02)	No causal association between psoriasis and fracture.
	Psoriatic arthritis (PsA)	eBMD	25 SNPs	301 667; European	One-sample MR	SD change in BMD per odds in log-odds of psoriatic arthritis susceptibility	0.88	heel BMD, 0.002 (−0.025–0.030)	No causal association between psoriatic arthritis and heel BMD.
				PsA: 3 061 cases, 13 670 controls eBMD: 462 824; European	Two sample MR		0.69	heel BMD, −0.001 (−0.005–0.003)	
	Psoriatic arthritis	Fracture	25 SNPs	PsA: 3 061 cases, 13 670 controls Fracture: 45 087 cases, 317 775 controls; European	Two sample MR	OR of fracture per doubling in odds of psoriatic arthritis susceptibility	0.52	Fracture, 0.99 (0.98–1.01)	No causal association between psoriatic arthritis and fracture.

*IVs* instrument variables, *25OHD* 25-hydroxyvitamin D, *2SLS* two-stage least squares, *BMC* bone mineral content, *BMD* bone mineral density, *BMI* body mass, *CAD* Coronary Artery Disease, *CI* confidence interval, *CRP* C-reactive protein, *DBP* vitamin D binding protein, *eBMD* estimated bone mineral density from ultrasound, *FA* forearm, *FHS* Framingham Heart Study, *FN* femoral neck, *IVW* Inverse-variance weighted, *IW* Instrumental variable, *LL* lower limbs, *LS* lumbar spine, *NA* not available, *OR* odds ratio, *PE* pelvis, *SD* standard deviation, *SK* Skull, *T1D* type 1diabetes, *T2D* type 2 diabetes, *TB* total body, *TSH* Thyroid Stimulating Hormone, *UL* upper limbs

#### Vitamin D and BMD/fracture

Vitamin D, by improving intestinal calcium absorption, has pivotal roles in bone heath. Vitamin D insufficiency was reported as a risk factor for several common diseases and conditions, including osteoporosis and osteoporotic fracture.^83^ However, the influence of vitamin D on the etiology of low bone mass and osteoporosis is unclear due to inconsistent results from clinical studies.^79^ Leong et al.^84^ investigated the causal relationship between vitamin D-binding protein (DBP) levels and BMD in the Canadian Multicentre Osteoporosis Study (CaMos) using individual-level data, and the results demonstrated a strong causal relationship between serum DBP and 25OHD levels; however, serum DBP had no causal effect on femoral neck BMD or osteoporosis (Table 2). Furthermore, Li et al.^85^ found no evidence for a causal effect of vitamin D levels on BMD (total hip, FN and LS) in Chinese postmenopausal women using four SNPs, *GC*-rs2282679, *NADSYN1*-rs12785878, *CYP2R1*-rs10741657 and *CYP24A1*-rs6013897, as candidate instrumental variables in the MR analyses. Recently, using data from the GEFOS consortium and UK Biobank, Larsson et al.^86^ found that vitamin D levels had no effect on BMD (FN, LS, heel) (*N* = 32 965). Recently, a study also showed a lack of a causal relationship between vitamin D levels and fracture risk by using 37 857 cases and 227 116 controls from the GEFOS Consortium, UK Biobank, EPIC-Norfolk study and 23andMe^36^ (Fig. 3, Panel B and Table 2). Similarly, as a provider of protein, micronutrients and dairy calcium, milk was recommended by some dietary guidelines, particularly for bone health. However, MR studies using a SNP (rs4988235) located upstream of the lactase gene as an instrumental variable found that milk consumption had no causal effect on BMD^87^ or fracture^36^ (Fig. 3, Panel B and Table 2).

#### Diseases and BMD/fracture

To date, diseases such as type 2 diabetes (T2D) and inflammatory diseases have been studied for their effects on osteoporosis or fracture (Table 2). Trajanoska et al.^36^ found that IBD was not a causal factor for fracture risk in 185 057 cases and 377 191 controls. More recently, with 432 513 samples from the UK Biobank dataset, Xia et al. found that psoriatic arthritis might be a risk factor for low BMD, but the link was not genetically determined. Psoriasis without arthritis is not a risk factor for osteoporosis.^88^

By using SNPs as IVs [32 SNPs strongly associated with type 2 diabetes (T2D), 30 SNPs associated with fasting glucose and 4 SNPs associated with 2-h glucose (2hGlu)], Ahmad et al.^89^ found that a genetically increased risk of T2D and a genetically increased risk of fasting glucose both had weak effects on increasing femoral neck BMD, but no significant trends were observed for the effect of T2D and fasting glucose on lumbar spine BMD.^89^ Furthermore, Trajanoska et al.^36^ found that T2D and fasting glucose were not causal for fracture in 185 057 cases and 377 191 controls. The study also reported no causal effect of type 1 diabetes (T1D) and coronary artery disease (CAD) on fracture.^36^

#### Other factors and BMD/fracture

Fat mass might be a causal decisive factor of bone mass, but the evidence was contradictory.^90–93^ By using variants of two loci [FTO (fat mass and obesity-associated gene) and MC4R (melanocortin 4 receptor)] strongly associated with fat mass and obesity, Timpson et al. evaluated the relation between fat mass and bone outcomes in ~5 000 children at a mean age of 9.9 years from the Avon Longitudinal Study of Parents and Children (ALSPAC) cohort and suggested that fat mass was the causal pathway for bone mass in children.^94^ In 2016, a study investigated whether adiposity was causal for BMD at the skull, upper limbs and lower limbs, pelvis and lumbar spine in 5 221 children from ALSPAC using 32 SNPs (strongly associated with BMI), and the results suggested that adiposity was causally related to increased BMD at all sites except the skull.^95^ The relationship between obesity and BMD was also investigated in adults, and it was found that obesity might be causally related to BMD at the femur but not at the spine.^96^ In addition, the MR approach has been used to show a positive causal association between serum estradiol concentrations and femoral neck BMD, lumbar spine BMD and heel BMD.^86^ Other studies demonstrated that earlier menopause and late puberty were causal factors for increasing fracture risk.^36,97^ However, urate,^78,98^ thyroid stimulating hormone (TSH),^36,99^ homocysteine,^36^ alcohol consumption^100^ and smoking status^36,100^ were not identified as causal factors for BMD or fracture by the MR approach (Fig. 3, Panel B). Notably, it was demonstrated that genetically decreased BMD was the only clinical risk factor with evidence for an effect on fracture risk among 15 clinically identified fracture factors.^36^ More recently, Cerani et al. undertook an MR study and found that a standard deviation increase in genetically derived serum calcium (0.13 mmol·L^−1^ or 0.51 mg·dL^−1^) was not associated with increased estimated BMD (426 824 subjects, *P* = 0.92) or a reduced risk of fractures (76 549 cases and 470 164 controls; *P* = 0.85).^101^

### Therapeutic targets for osteoporosis

Despite the small effect size of common variants identified by GWASs, most of the osteoporosis agents in use (or undergoing trials) target pathways related to the GWAS-discovered BMD genes, and genetic information might significantly improve the search for drug targets and increase the success rate of preclinical and clinical trials.^102^ Moreover, it is well recognized that the effect size of association is not well correlated with clinical relevance, as many FDA-approved medications target proteins linked to common variants identified by GWASs.^102–104^ An example of success in the field was the use of GWAS data for drug repositioning studies. Sanseau et al.^105^ found that among the publicly relevant disease-related GWAS loci, 155 out of the 991 loci (15.6%) were related to drug development. Among them, the drug indications of 63 targeted proteins matched the corresponding GWAS traits, indicating that the pathogenic genes excavated by GWASs had a higher probability of being directly used as drug targets.^105^ For example, the *IL12B* (interleukin 12B) gene found in psoriasis GWASs encodes the target of ustekinumab, a newly proven drug for psoriasis. In addition, the gene was considered to be related to Crohn’s disease, and the development of related drugs was in a phase II clinical trial.^105^ Another example was denosumab, which is a drug marketed for the treatment of osteoporosis in postmenopausal women, targeting the gene *TNFSF11* (tumor necrosis factor superfamily, member 11), also known as *RANKL*. Denosumab is a RANKL inhibitor that functions by preventing the development of osteoclasts. Recently, it was speculated that the drug might have a therapeutic effect on Crohn’s disease, as *TNFSF11* was found to be significantly associated with Crohn’s disease in GWASs.^106^ The current drugs that are available for the treatment of osteoporosis and their most likely targets are listed in Table 3. Five anti-osteoporosis therapeutics currently approved or in advanced clinical trials were supported by GWAS data. It was reasonable to believe that the findings of GWASs could be potentially powerful in the identification of anti-osteoporosis drug targets and drug repositioning.

**Table 3:** Present and potential near-term osteoporosis drug targets that have been linked to changes in BMD by GWAS. Table adapted from^178^

Drug class	Drug target	Principle	Stage	Target locus identified through GWASs	Refs.
Denosumab	RANKL	Reduces bone resorption by selectively targeting RANKL	Approved for clinical use	RANKL	^179^
Sclerostin inhibitors (Romosozumab)	Sclerostin (SOST)	Improve the recruitment and activation of osteoblasts by targeting Wnt/β-catenin signaling pathways	Approved for clinical use in Japan, US and Europe	SOST	^107^
Selective estrogen receptor modulators	Estrogen receptor	Reduces bone resorption	Approved for clinical use	ESR1	^180^
		by targeting the OPG/RANK/RANKL pathway			
Parathyroid hormone analogs	Parathyroid hormone receptor	Majorly participate in the process of bone formation	Approved for clinical use	Not identified, but the pathway has been	^181,182^
		by targeting the PKA pathway		highlighted through PTHLH (encodes PTHRP)	
Bisphosphonates	Farnesyl pyrophosphate	Inhibition of bone resorption	Approved for clinical use	Not identified	^183^
Estrogen ESR1	Estrogen receptor	Reduces bone resorption	Approved for clinical use	ESR1	^184^
		by targeting the OPG/RANK/RANKL pathway			
Cathepsin K inhibitors	Cathepsin K	Inhibition of bone resorption	Terminated	Not identified	^185^
		by targeting the OPG/RANK/RANKL pathway			
DKK1 inhibitors	DKK1	Improve bone formation by targeting the Wnt/β-catenin signaling pathway	In the preclinical phase	DKK1	^120^

*DKK1* dickkopf 1, *ESR1* estrogen receptor 1, *OPG* osteoprotegerin, *PKA* protein kinase A, *PTHLH* parathyroid hormone-like hormone, *PTHRP* parathyroid hormone-related protein, *RANK* receptor activator of nuclear factor kβ, *RANKL* RANK ligand, *SOST* sclerostin, *US* United States

The *SOST* (sclerostin) gene was found to be strongly associated with BMD by GWASs;^107^ *SOST* produces sclerostin, which is a key Wnt pathway regulator that is preferentially expressed by osteocytes. Sclerostin acts by binding to the Wnt coreceptor LRP5/6 by competing with Wnt protein; as a consequence, sclerostin blocks the accumulation of β-catenin in the cytoplasm, inhibits the differentiation and proliferation of osteoblasts, enhances osteoclastogenesis and causes bone loss.^108,109^ Given the inhibitory effect of sclerostin on osteoblast function and bone formation, blocking the activity of sclerostin to activate this pathway seems to be a potential strategy in the treatment of osteoporosis. Romosozumab (AMG785/CDP-7851), a monoclonal humanized antibody to sclerostin, was evaluated for its efficacy. Compared with the traditional bone resorption inhibitor alendronate and the bone formation promoter teriparatide, the greatest feature of romosozumab was its ability to reverse postmenopausal osteoporosis in women with hormone deficiency.^110–112^ Saag et al. compared the effect between romosozumab (210 mg monthly administered subcutaneously) and alendronate (70 mg weekly) for 12 months, followed by open-label alendronate 70 mg weekly for another 12 months in postmenopausal women with osteoporosis and a fragility fracture^113^. After 24 months, a lower risk of fractures, including clinical fractures (27% lower), hip fractures (38%), new vertebral fractures (48% lower) and nonvertebral fractures (19%), was observed in the romosozumab-to-alendronate group than in the alendronate-to-alendronate group.^113^ A phase III clinical trial was conducted to estimate the effect of romosozumab (*n* = 206) versus teriparatide (*n* = 209) on osteoporosis in postmenopausal women who took oral bisphosphonate for at least 3 years, and it was found that romosozumab (210 mg once monthly) had a greater effect on hip BMD than subcutaneous teriparatide (20 μg once daily)^114^. Another trial recruited 7 180 postmenopausal women who had osteoporosis, and the subjects were randomly assigned to receive subcutaneous injections of romosozumab (at a dose of 210 mg) or placebo monthly for 12 months; thereafter, both groups received denosumab 60 mg every 6 months twice.^115^ At the end of the initial 12 months, romosozumab had decreased the incidence of new vertebral fractures and nonvertebral fractures by ~73% and 24%, respectively.^115^ At 24 months, a 75% lower risk of vertebral fractures was seen in the romosozumab group after the transition to denosumab.^115^

On 9 April 2019, the US Food and Drug Administration (FDA) approved romosozumab for the treatment of osteoporosis in postmenopausal women at high risk of fracture, with a boxed warning highlighting the risk of cardiovascular adverse events and a postmarketing requirement to assess the cardiovascular safety of romosozumab.^116^ On 28 June 2019, the European Medicines Agency (EMA) recommended the refusal of the marketing authorization for Evenity (romosozumab) because the results suggested that patients given Evenity had an increased risk of serious effects on the heart and circulatory system, such as heart attacks or strokes.^117^ In addition, there were more deaths in patients aged over 75 years who were given the medicine. As it was unclear why the medicine appeared to increase the risk of heart and circulatory problems, measures to reduce the risk could not readily be put in place.^117^ Dramatically, after re-examining initial opinions, the EMA noted that the medicine showed convincing evidence of benefit in women with severe osteoporosis, with better effect than alendronate, and it was suggested that only women who had no history of heart attack and stroke could take the medicine.^117^ On 17 October 2019, the EMA recommended that marketing authorization be granted but for a restricted indication in postmenopausal women with severe osteoporosis at high risk of fracture.^117^

Dickkopfs (DKKs) are secreted proteins composed of two cysteine-rich domains with four homologous forms (DKK-1~4) in vivo. DKK-1 inhibited the Wnt/β-catenin signaling pathway by directly binding to LRP5/6 and formed a complex with Kringen, a transmembrane protein containing a Kringle domain, which increased endocytosis and decreased LRP5/6 content, thus leading to the inactivation of the Wnt pathway.^118,119^ DDK1 is closely related to bone mass,^120,121^ and similar to sclerostin monoclonal antibodies, monoclonal antibodies to DKK-1 increase trabecular mass and density in mice^122^ and restore bone density in osteoporotic mice and rhesus monkeys.^123^ Monoclonal antibodies to DKK-1 included BHQ880^124^ and PF04840082,^125^ but both were in the preclinical phase.

### Prediction of osteoporosis and fracture

One of the goals of genetic study is to improve the value of clinical application, for example, to predict osteoporosis or fracture risk from GWAS findings. Studies have shown that at least 150 loci with an OR value of 1.5 or 250 loci with an OR value of 1.25 were required for the prediction of disease risk.^126^ This suggested that any single locus could not be useful in clinical prediction, regardless of the size of the effect. However, theoretical and empirical studies have suggested that profiling multiple variants that are associated with bone phenotypes could improve the accuracy of fracture prediction and classification beyond that obtained by conventional clinical risk factors, such as the Fracture Risk Assessment Tool (FRAX).^127^

#### Polygenic risk scoring

Polygenic risk scoring was one primary approach for disease risk prediction. In a semisimulation study for fracture, it was shown that a profiling of up to 25 genes/variants (each with a relative risk of 1.10–1.35 and frequency ranging from 0.25 to 0.60) in the presence of clinical risk factors—with or without BMD—could achieve an AUC of 0.80.^128^ Ho-Le et al. took 62 BMD-associated SNPs to define the predictive value of genetic profiling for fracture prediction in 557 men and 902 women and found that individuals with a greater polygenic risk score (PRS) had a lower femoral neck BMD (*P* < 0.01); each unit increase in PRS was associated with a hazard ratio of 1.20 for fracture, and this association was independent of age, prior fractures and falls.^129^. However, polygenic risk scoring remained limited due to the linkage disequilibrium (LD) pruning of SNPs (prioritizing the most significant associations up to an empirically determined *P* value threshold, and pruning the SNPs based on LD).^130^ To remediate this issue, recent developments in machine learning may be a novel strategy.^131,132^ Machine learning approaches adapted a set of sophisticated statistical and computational algorithms to make predictions by mathematically mapping the complex associations between a set of risk SNPs to complex disease phenotypes.^133^ The optimal predictive ability for the target disease was obtained by mapping the pattern of selected features in the training genotype data, and at the end of the training stage, the model with the maximum predictive ability of the training dataset was selected for validation.^131,134^ Machine learning has been applied to the prediction of diseases or traits, such as inflammatory bowel disease,^135^ Alzheimer’s disease,^136^ cancers,^137,138^ heart failure^139^ and height.^140^

#### Machine learning methods

Through the analysis of 341 449 individuals from the UK Biobank, Forgetta et al. tested whether machine learning methods could provide a clinically relevant genomic prediction of quantitative ultrasound speed of sound (SOS)—a risk factor for osteoporotic fracture.^141^ In the Model Selection Set, age, sex and BMI explained 4.0% of the variance in SOS; the addition of the remaining FRAX clinical risk factors increased the variance explained to 4.8%, whereas when polygenic risk scores across different *P* value thresholds were added, the variance explained increased to at most 18.5%.^141^ Surprisingly, the machine learning algorithm improved the explained variance in SOS to a maximum of 25.0%. Then, they selected the top model (the machine learning algorithm selected 21 717 activated SNPs with a *P* value ≤ 10^−4^) from the Model Selection Set to test for its correlation with the SOS in the validation set and found that the model could explain 23.2% of the variance in the measured SOS. Subsequently, they evaluated the associations among SOS, genomically predicted SOS (gSOS), BMD and fracture and found that decreased SOS and fracture were both strongly associated with increased odds of incident fracture (gSOS had the highest risk per SD) in the univariate model. However, in multivariate models, gSOS was more strongly associated with major osteoporotic fracture than SOS or BMD.^141^ For fracture prediction, gSOS outperformed FRAX clinical risk factors alone. The machine learning algorithms provided better predictions than traditionally used polygenic risk scores. These findings suggested that genetic profiling of BMD-associated genetic variants could improve the accuracy of fracture prediction over and above that of clinical risk factors alone.

### Perspective

Despite fruitful GWAS discoveries in the bone field, most of these GWAS participants were of European descent. In fact, if we extended to other complex traits, ~79% of GWASs were conducted in European populations according to the GWAS catalog. Martin et al.^142^ systematically evaluated the polygenic risk prediction accuracy in Japanese, British and African-descent individuals on the basis of using independent GWASs of equal sample sizes from BioBank Japan (BBJ) and UK Biobank, including 17 quantitative anthropometric and blood panel traits and five disease endpoints; they demonstrated that prediction accuracy was consistently higher with GWAS summary statistics from ancestry-matched summary statistics. The condition of genetic resources and analyses overwhelmingly centered on individuals of European ancestry would lead to imbalances in the subsequent translatability of findings. To realize the full and equitable potential of the polygenic risk score, it was encouraged that more GWASs and sequencing studies on osteoporosis, BMD and fracture should be carried out in additional ethnic populations, such as the Chinese population, which made up ~20% of the global population. Fortunately, the cost of whole-genome sequencing and genotyping has dramatically decreased, making the utility of genetic variants more affordable and practical. In addition, the prioritization of the recruitment and analysis of diverse cohorts would become smooth with an increasingly globalized and connected research community.^143^

In summary, the achievement of GWASs is unprecedented in the understanding of how genetic variants influence osteoporosis and fracture. In the future, by mining large databases with detailed characterization of relevant phenotypes, more causal genes/mutations will be identified. In addition, large-scale genetic data could provide a new way to identify new drug targets and could be translated into precision treatment options to prevent and treat osteoporosis and fracture.

## Supplementary information

Box 1 Introduction of Genome-Wide Association Studies

Box 2 Introduction of Mendelian Randomization (MR)

Supplementary table 1
