# Supplementary material for: Twelve years of GWAS discoveries for osteoporosis and related traits: advances, challenges and applications
Source: Bone Res. 2021 Apr 29;9:23. doi: 10.1038/s41413-021-00143-3 (PMC8085014; doi:10.1038/s41413-021-00143-3)
Supplement: Supplementary file 1 — Box 1 Introduction of Genome-Wide Association Studies [file 41413_2021_143_MOESM1_ESM.docx]

**Box 1 Introduction of Genome-Wide Association Studies (GWASs)**

**(1) Principle**

GWASs represent a valuable approach to type hundreds of thousands of single-nucleotide polymorphisms (SNPs) to investigate the associations between genetic variants and traits, including diseases.

**(2) SNPs and Genetic Variation**

SNPs are an area of DNA that varies in a single nucleotide or base pair, which are only one type of genetic variation found in the genome but are the most common. There are only four bases, A (adenine), C (cytosine), G (guanine), and T (thymine). GWASs look for these specific loci or SNPs in the entire genome to see whether some are more common in people with a particular trait or disease.

**(3) Genotypes and Phenotypes**

Genotypes refer to genetic variations, such as variations in SNPs. Phenotypes refer to traits (for example, height or BMD) but may also include diseases (for example, obesity, osteoporosis or fracture).

**(4) How to perform GWASs?**

When looking at common medical conditions (such as osteoporosis), researchers recruit one group of people with the disease and another group without the disease. The frequency of the SNPs is compared between these two groups to see whether there are any associations between genotype (genetic variations, such as variations in SNPs) and the phenotype (the disease).
