# Supplementary material for: Twelve years of GWAS discoveries for osteoporosis and related traits: advances, challenges and applications
Source: Bone Res. 2021 Apr 29;9:23. doi: 10.1038/s41413-021-00143-3 (PMC8085014; doi:10.1038/s41413-021-00143-3)
Supplement: Supplementary file 2 — Box 2 Introduction of Mendelian Randomization (MR) [file 41413_2021_143_MOESM2_ESM.docx]

**Box 2 Introduction of Mendelian Randomization (MR)**

**(1) Categories of Mendelian randomization analysis^1,2^**

*Single-sample MR* uses genetic instruments, exposure, and outcome in the same sample.

*Two-sample MR* estimates causal effects from data on the exposure and outcome from two (or only partially overlapping) samples.

*Bidirectional MR* is performed in one direction (“exposure” to “outcome”) and then in the opposite direction (“outcome” to “exposure”) using the SNPs strongly associated with each trait in the independent GWAS.

*Two-step MR* assesses whether an intermediate trait acts as a causal mediator between exposure and outcome. In the first step, genetic instruments for the exposure are used to estimate the causal effect of exposure on the mediator. In the second step, genetic instruments for mediators are used to assess the causal effect of the mediator on outcome.

*Multivariable MR* uses genetic instruments associated with multiple exposures to estimate the independent causal effect of the risk factors on the outcome.

**(2) Statistical methods^2,3^**

*Inverse variance weighted (IVW)*—IVW MR is equivalent to a weighted linear regression of SNP-outcome associations on SNP exposure.

*Weighted median* is defined as the median of the weighted empirical density function of the ratio estimates that allows up to 50% of the information in MR analysis to come from invalid instruments.

*Mode-based estimate (MBE)* provides a consistent estimate of the causal effect if the most common pleiotropy value across instruments is zero.

*MR-Egger* is not restrained to have a slope by way of zero; hence, its causal estimate shows a genotype-outcome dose-response relationship.

*Likelihood-base method* assumes a linear relationship between exposure and outcome and uses a bivariate normal distribution for the genetic estimates.

**(3) Method for explore and adjust for pleiotropy^2-6^**

*Intercept of the MR-Egger regression* can test the average pleiotropic effect across all genetic variants and provide an estimate of the causal effect of exposure on outcome.

*Cochran’s Q-statistic (IVW)* can test for heterogeneity between genetic instruments used that could indicate pleiotropic effects.

*Pleiotropy-robust MR (PPMR)* provides an unbiased causal estimate in the presence of pleiotropy by subtracting the pleiotropic effect (of an instrument) estimated in a subgroup of the population for whom the instrument is not associated with the exposure.

*Weighted median estimator* can provide reliable evidence as long as at least half of the genetic variants have no pleiotropic effects.

*Mendelian randomization pleiotropy residual sum and outlier (MR-PRESSO)* can evaluate horizontal pleiotropy and test the significant differences in the causal estimates before and after the removal of outliers.

**References**

1. Burgess, S., Butterworth, A. & Thompson, S. G. Mendelian randomization analysis with multiple genetic variants using summarized data. Genet. Epidemiol. 37, 658–665 (2013).

2. Davies, N. M., Holmes, M. V. & Davey Smith, G. Reading Mendelian randomization studies: a guide, glossary, and checklist for clinicians. Bmj 362, k601 (2018).

3. Bowden, J., Davey Smith, G. & Burgess, S. Mendelian randomization with invalid instruments: effect estimation and bias detection through Egger regression. Int. J. Epidemiol. 44, 512–525 (2015).

4. Bowden, J. et al. A framework for the investigation of pleiotropy in two-sample summary data Mendelian randomization. Stat. Med. 36, 1783–1802 (2017).

5. Zheng, J. et al. Recent developments in mendelian randomization studies. Curr. Epidemiol. Rep. 4, 330–345 (2017).

6. Bowden, J., Davey Smith, G., Haycock, P. C. & Burgess, S. Consistent estimation in Mendelian randomization with some invalid instruments using a weighted median estimator. Genet. Epidemiol. 40, 304–314 (2016).
